# Primary Retroperitoneal Mucinous Cystadenocarcinoma in a Male Patient: A Case Report

**DOI:** 10.3390/curroncol32090500

**Published:** 2025-09-05

**Authors:** Masayuki Tomioka, Keita Nakane, Koji Iinuma, Kota Kawase, Tomoki Taniguchi, Yuki Tobisawa, Aoi Muto, Tomohiro Kanayama, Tatsuhiko Miyazaki, Takuya Koie

**Affiliations:** 1 Department of Urology, Gifu University Graduate School of Medicine, Gifu 5011194, Japan; tomioka.masayuki.e4@f.gifu-u.ac.jp (M.T.); nakane.keita.k2@f.gifu-u.ac.jp (K.N.); iinuma.koji.s0@f.gifu-u.ac.jp (K.I.); kawase.kota.b5@f.gifu-u.ac.jp (K.K.); taniguchi.tomoki.a8@f.gifu-u.ac.jp (T.T.); tobisawa.yuki.a7@f.gifu-u.ac.jp (Y.T.); 2 Department of Pathology, Gifu University Hospital, Gifu 5011194, Japan; muto.aoi.i2@f.gifu-u.ac.jp (A.M.); kanayama.tomohiro.u7@f.gifu-u.ac.jp (T.K.); miyazaki.tatsuhiko.z0@f.gifu-u.ac.jp (T.M.)

**Keywords:** primary retroperitoneal mucinous cystadenocarcinoma, retroperitoneal tumor, male patient

## Abstract

Primary retroperitoneal mucinous cystadenocarcinoma (PRMC) is an uncommon malignant neoplasm with few reported cases, particularly among male patients. Currently, only nine documented cases have been reported worldwide, including the present case. The present case report describes the incidental detection of PRMC in an 86-year-old male patient. Despite being offered surgical intervention, the patient initially opted against treatment. Consequently, follow-up imaging examinations were performed for 3 subsequent years. The tumor, initially measuring 31 × 32 × 31 mm, gradually increased to 58 × 60 × 59 mm. Subsequently, the patient underwent laparoscopic retroperitoneal tumor resection. Histopathological examination revealed adenocarcinoma characterized by intestinal differentiation. The patient has exhibited no evidence of disease for 1 year postoperatively. The present case is noteworthy, as this disease rarely occurs in men, thereby offering significant potential for educational and scientific contributions. Notably, the patient’s age, longitudinal observation of tumor progression through imaging over a period of 3 years, and complete surgical excision of the tumor are salient features of this case. These findings may prove useful in the diagnosis and treatment strategy for male patients with PRMC.

## Simple Summary

Herein, we present the case of a man with an extremely rare primary retroperitoneal mucinous cystadenocarcinoma. After 3 years of follow-up with imaging examinations, the patient underwent laparoscopic resection.

## 1. Introduction

Histopathological analysis of primary retroperitoneal mucinous tumors classifies them into three distinct categories: mucinous cystadenomas, mucinous borderline tumors, and mucinous cystadenocarcinoma [1]. Primary retroperitoneal mucinous adenocarcinoma (PRMC) is a prevalent tumor type; however, its development as a primary tumor in the retroperitoneum is uncommon [1,2]. Eighty cases that have been documented in the English literature are distinguishable from benign cystadenomas and borderline malignant tumors and mostly occurred in women [1]. The increased prevalence of PRMC among women is primarily due to the presence of heterotopic ovarian tissue, which is the primary site of origin of this neoplasm [3,4]. Consequently, PRMC’s occurrence in male patients is exceptionally rare, with only eight documented cases reported to date [2,3,4,5,6,7,8]. The etiology, biological characteristics, and prognosis of this tumor remain unclear because of its rarity [2,9]. Moreover, accurate preoperative diagnosis is often challenging, and optimal treatment remains to be elucidated [9]. Herein, we present the ninth documented case worldwide involving surgical intervention following prolonged follow-up observation after tumor identification in a man. This case marks a significant milestone in the field, as this is the first instance in which the natural progression of PRMC in a man was successfully tracked for 3 years using radiographic examinations. Moreover, this case involves the oldest male patient reported to date and is the first in which tumor control was achieved through surgical treatment.

## 2. Case Description

**Figure 1:**
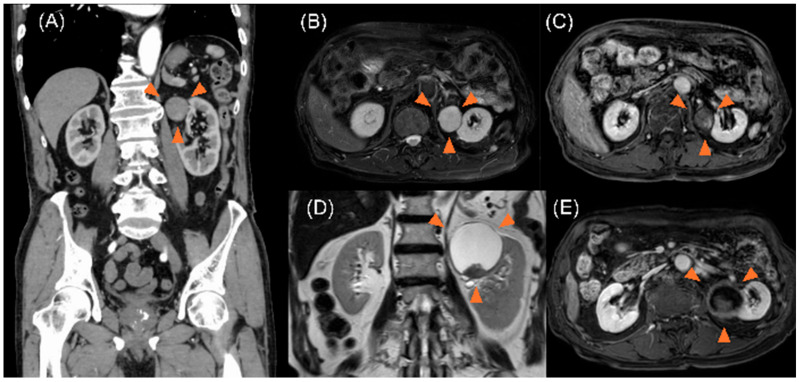
(**A**) Contrast-enhanced computed tomography images showing a cystic lesion (31 × 32 × 31 mm) containing partial solid components in the upper pole of the left kidney (arrow). (**B**) T2-weighted magnetic resonance imaging showing the presence of cystic lesions, exhibiting high intensity (arrow). (**C**) Contrast-enhanced T1-weighted magnetic resonance imaging showing the presence of solid components with uneven contrast enhancement within the cystic tumor (arrow). (**D**) Magnetic resonance imaging performed three years later showing a cystic lesion exhibiting enlargement to 58 × 60 × 59 mm on T2-weighted images (arrow). (**E**) T1-weighted magnetic resonance imaging study showing an enlarged cystic lesion, accompanied by the presence of solid components within the lesion (arrow).

An 86-year-old male patient with a history of gastric ulcers and hypertension was admitted to the Department of Dermatology for burn treatment. A left retroperitoneal tumor was incidentally identified on computed tomography (CT). Consequently, the patient was referred to the Department of Urology for further evaluation. Contrast-enhanced CT and magnetic resonance imaging revealed a cystic lesion (31 × 32 × 31 mm) with solid components near the upper pole of the left kidney (Figure 1A–C). The decision to proceed with initial surveillance was based on the following factors: (1) the patient was 86 years of age and had multiple comorbidities, (2) the lesion was asymptomatic, (3) the initial lesion was small, with no clear evidence of progressive characteristics, and (4) the patient strongly desired to avoid surgery at that time after receiving comprehensive counseling on the risks and benefits. Subsequent imaging examinations were performed at 3–4-month intervals to monitor tumor progression. However, the tumor gradually increased in size and reached 58 × 60 × 59 mm approximately 3 years later (Figure 1D,E).

**Figure 2:**
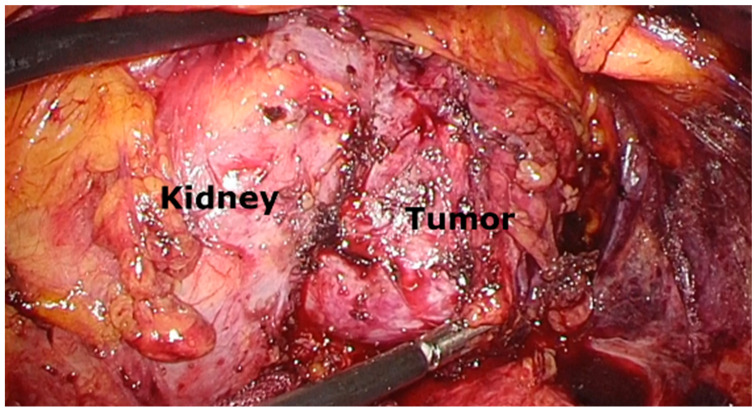
Image during laparoscopic retroperitoneal tumor resection showing adhesions between the tumor and the left kidney. However, the resection surface is easily identifiable, enabling complete resection of the tumor alone.

Owing to the enlargement of the cystic neoplastic lesion, the patient ultimately underwent laparoscopic retroperitoneal tumor resection. The total operative time was 216 min, with an estimated blood loss of 30 mL. During the surgical procedure, mild adhesions were observed between the tumor and the kidney; however, the resection surface was easily identified (Figure 2). The tumor was completely extirpated without compromising cyst wall integrity. The postoperative course was uneventful. The patient was able to walk on postoperative day 1 and was discharged on postoperative day 7. No surgery-related complications were observed during the 30-day postoperative period.

**Figure 3:**
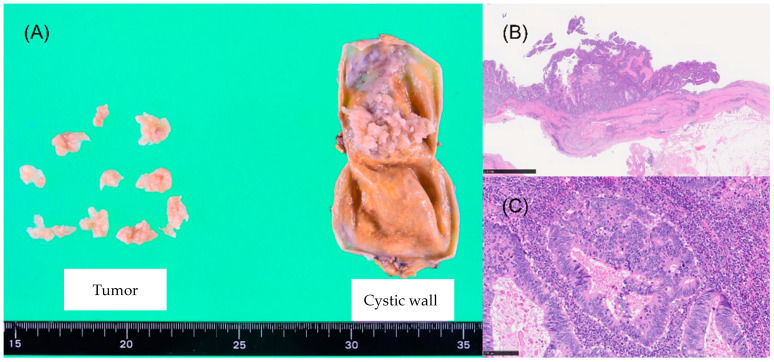
(**A**) The macroscopic findings of the resected specimen reveal significant findings. The presence of a papillary substance is visible on the inner surface of the divided cyst wall on the right side. The left side of the image displays fragments of the internal components of the tumor. (**B**) The histopathological findings, based on hematoxylin and eosin staining of the resected tumor, reveal the presence of cyst walls and papillary components within the cyst (4× magnification). (**C**) Within the papillary region, atypical glandular cells appear to have proliferated in fused tubular-to-tubular and papillary patterns, accompanied by dense inflammatory infiltrates and numerous mitotic figures (hematoxylin and eosin staining, 40× magnification).

Macroscopic examination revealed solid papillary components in the cystic tumor (Figure 3A). Histological analysis indicated a single layer of cuboidal to flat epithelial cells, despite the majority of the cyst wall exhibiting signs of cell desquamation. Papillary tumors were identified based on the presence of atypical glandular cells forming tubular and papillary structures, numerous nuclei with mitotic activity, and inflammatory cell infiltration (Figure 3B,C).

Postoperatively, the serum carcinoembryonic antigen (CEA) levels were recorded at 19.9 ng/mL, while carbohydrate antigen 19-9 (CA19-9) levels were 6.1 U/mL. Subsequent monitoring revealed that both markers had returned to normal levels within three months after surgery. One year after the surgical procedure, there were no indications of recurrence, thereby maintaining a state of no evidence of disease.

## 3. Discussion

PRMC is an extremely rare malignant neoplasm that occurs in the retroperitoneal cavity without clear primary lesions in adjacent organs, such as the pancreas, colon, and kidneys [6]. Retroperitoneal tumors account for approximately 0.1–0.2% of all cancers; however, PRMCs account for only a small proportion of all retroperitoneal neoplasms, with approximately 80 cases reported to date [1,10,11]. PRMCs are predominantly observed in women owing to their etiology, which originates from the heterotopic ovarian tissue [1]. Conversely, only nine cases have been documented in men, including the present case (Table 1). The age of the males who developed PRMC ranged from 30 to 83 years, and they did not manifest symptoms characteristic of PRMC, such as chronic back pain, abdominal pain, sensations of fullness, or weight loss [2,3,4,5,6,7,8,11]. This patient was previously diagnosed with PRMC at 86 years of age, making him the oldest reported male patient with the disease. Although tumors > 10 cm are sometimes diagnosed, there are cases, such as the present case, in which small lesions are discovered incidentally or during detailed examinations for minor symptoms [2,6]. Imaging examinations often recognize PRMCs as multicystic retroperitoneal tumors located away from major organs; therefore, they can be differentiated from organ-derived malignant tumors [8,12]. Although there are documented cases of elevated levels of tumor markers, such as CEA and CA19-9, there are also reports of normal levels. Consequently, the diagnosis of PRMC based solely on tumor markers is constrained [8,12]. Therefore, PRMC should be considered as a differential diagnosis, as it is a rare disease with the potential to affect older adult males.

Differential diagnosis for PRMC encompasses various etiologies, including metastatic mucinous adenocarcinoma from gastrointestinal or gynecological malignancies, primary retroperitoneal sarcoma with cystic degeneration, malignant lymphoma, and paraganglioma. Additionally, the differential diagnosis should include benign lesions, such as lymphangioma, teratoma, pancreatic pseudocysts, and infectious cystic lesions [1,7]. The fundamental aspects of PRMC diagnosis are as follows: the primary organ is difficult to identify, complex cystic tumors and solid components coexist, and the tumor is a mucinous adenocarcinoma with intestinal-type epithelium [1]. Histological evaluation is necessary to ensure an accurate diagnosis of PRMC. However, considering the absence of characteristic imaging findings and PRMC-specific tumor markers, obtaining an accurate preoperative diagnosis is challenging. Additionally, PRMC may be difficult to differentiate from renal cysts and tumors in imaging examinations owing to its high similarity to these structures [12,15].

A noteworthy point in this case was the period of preoperative radiological monitoring. The lesion was discovered incidentally and closely monitored using imaging tests every 3–4 months for 3 years. During the observation period, the tumor size increased from 31 × 32 × 31 mm to 58 × 60 × 59 mm, with an annual growth rate of approximately 1 cm. To the best of our knowledge, no reports exist on long-term imaging-based observations preoperatively in men with PRMCs. The results demonstrate the importance of careful follow-up imaging if malignant neoplasms, including PRMC, are suspected. Recent findings indicate that positron emission tomography-CT is crucial in differentiating malignant and benign retroperitoneal cystic lesions, and fluorodeoxyglucose uptake patterns may be useful for preoperative diagnosis [16].

The prevailing treatment modality for PRMCs involves complete surgical resection, which is considered the most effective curative approach. In the context of PRMCs in male patients, reports of complete surgical resection have demonstrated no recurrence and favorable prognoses [2,6,8,12,17]. The extreme rarity of PRMC has resulted in the absence of an established chemotherapy regimen, and the treatment for ovarian mucinous carcinoma may be selected based on histopathological similarities [9]. The most prevalent chemotherapy regimen uses carboplatin, with an area under the curve of 6, and paclitaxel at 175 mg/m^2^ is administered every 21 days [9]. However, considering the lack of evidence supporting the efficacy of anticancer drug therapy, surgical resection may be the optimal treatment option for PRMC.

Although PRMC is a rare malignant neoplasm in men, meticulous treatment options for retroperitoneal cystic lesions are imperative, even in cases that are asymptomatic or grow slowly in the early stages. Particularly, the patient underwent a series of imaging procedures for 3 years, culminating in surgical intervention following the detection of a growth tendency. This observation underscores the importance of timely intervention, as PRMCs may progress insidiously over time, even in the absence of initial symptoms.

Several hypotheses have been proposed regarding the primary site of PRMC in men. Although PRMC is thought to originate from remnants of germ cells, disseminated Müllerian epithelium has been suggested as a possible cause in female patients [17,18]. Therefore, residual Müllerian and Wolffian duct tissue is also present in male patients, which may cause PRMC development [17,18]. Although the mesonephric gland has not been clearly identified in PRMC specimens, the theory that PRMC originates from remnants of the urogenital tract is still widely accepted [18,19]. Meanwhile, PRMC has also been proposed to arise from teratoma elements of unrecognized or treated germ cell tumors, particularly in cases with precursor germ cell tumors or paratesticular sites [19,20]. However, current research suggests that PRMC most likely originates from the peritoneal epithelium, which exhibits mucinous dysplasia [5,6,7]. This phenomenon is hypothesized to result from the attachment of coelomic epithelial cells derived from the urogenital ridge to the retroperitoneal region during embryonic development. These epithelial cells undergo differentiation into Müllerian ducts, which are believed to result in the formation of inclusion cysts. Subsequently, the body cavity epithelium within these cysts undergoes metabolic degeneration, progressing from benign to borderline malignant and clearly malignant cells [5,6,7]. Recently, several markers have emerged as potential indicators for diagnosing PRMC. *KRAS* and *GNAS* mutations, which are frequently observed in pancreatic and appendix mucinous neoplasms, have been documented to be positive in some cases of PRMC. To elucidate the pathophysiology of PRMC in men and establish effective treatment methods, the accumulation of cases and elucidation of molecular profiles may be necessary in the future [20].

The most probable hypothesis is that PRMC in men is of non-ovarian origin, and the residual peritoneal epithelium with Müllerian differentiation potential is the most probable site of origin. However, further molecular, biological, and immunohistochemical studies are needed to establish the cellular origin of male PRMC and elucidate their etiology. A limitation of this report is the relatively short follow-up period of 1 year postoperatively. Although no recurrence has been observed, long-term follow-up is essential considering the limited data on long-term prognosis in men with PRMC.

## Figures and Tables

**Table 1:** Published cases of primary retroperitoneal mucinous cystadenocarcinoma occurring in men.

Author, Year	Age (Years)	Tumor Size (cm)	Symptoms	Management	Outcome
Thamboo [5], 2006	64	24 × 20 × 16	Acute abdominal discomfort	Complete tumor excision (laparotomy)	No recurrence at 18 months
Green [2], 2007	83	26 × 20 × 16	Abdominal discomfort and cachexia	Complete tumor excision (open surgery)	No recurrence at 6 months
Hrora [6], 2009	42	5 × 4 × 3	Abdominal discomfort and distension	Two-stage surgical removal of all cysts	No recurrence at 6 months
Shiau [7], 2013	59	7.5 × 7 × 3	Sudden onset of left flank pain	Complete tumor excision (laparotomy)	No recurrence at 79 months
Feng [8], 2013	63	4 × 3 × 3	Chronic lower back pain	Complete tumor excision (laparotomy)	No recurrence at 13 months
Vargas [13], 2015	68	16 × 14.5 × 11	Self-detected palpable right abdominal mass	Complete tumor excision (laparotomy)	Not reported
Khurana [12], 2016	57	21 × 18 × 7	Weight loss, poor appetite, and palpable fixed mass	Complete tumor excision (laparotomy)	No recurrence at 15 months
Morihisa [14], 2023	60	20 × 8.8 × 4.7	Lower back pain	Systemic chemotherapy with carboplatin and paclitaxel	Bone metastasis at diagnosis
Present case	86	58 × 60 × 59	Asymptomatic	Complete tumor excision (laparotomy)	No recurrence at 12 months

## Data Availability

The original contributions presented in this study are included in the article. Further inquiries can be directed to the corresponding author.

## Abbreviations

The following abbreviations are used in this manuscript:

PRMC	Primary retroperitoneal mucinous adenocarcinoma
CT	Computed tomography
CEA	Carcinoembryonic antigen
CA	Carbohydrate antigen 19-9
CK	Cytokeratin
